# Anti-Aging Efficacy of Low-Molecular-Weight Polydeoxyribonucleotide Derived from *Paeonia lactiflora*

**DOI:** 10.3390/ijms27010220

**Published:** 2025-12-24

**Authors:** Sun-Uk Bak, Min Sook Jung, Da Jung Kim, Hee Un Jin, Seung Youn Lee, Chae Eun An

**Affiliations:** Skin Science Research Center, NewLife BST Co., Ltd., Seoul 08594, Republic of Korea; subak@newlifegroup.com (S.-U.B.); msjeong@newlifegroup.com (M.S.J.); hujin@newlifegroup.com (H.U.J.); syl@newlifegroup.com (S.Y.L.); cean@newlifegroup.com (C.E.A.)

**Keywords:** polydeoxyribonucleotide, Peony PDRN, *Paeonia lactiflora*, skin aging, extracellular matrix, low-molecular-weight, UV-induced damage, skin regeneration, skin barrier

## Abstract

Polydeoxyribonucleotide (PDRN), a DNA fragment mixture, exerts biological effects via adenosine A2A receptor and salvage pathway activation. Here, *Paeonia lactiflora*-derived PDRN (Peony PDRN) is proposed as a plant-based alternative to salmon-derived PDRN. While *P. lactiflora* is known for its medicinal properties, the biological functions of Peony PDRN have not been characterized. To validate and optimize its efficacy, we systematically compared the biological activities of three molecular weight groups of Peony PDRN (high, medium, and low) using in vitro assays and clinical studies. The low-molecular-weight fraction (Low-Peony PDRN) markedly enhanced skin cell proliferation and migration, upregulated extracellular matrix-related genes (*COL1A1*, *COL5A1*, *ELN*, and *FBN1*), and promoted keratinocyte differentiation and epidermal barrier formation by increasing *COL7A1*, *IVL*, *FLG*, and *OCLN* expression. It also reduced reactive oxygen species levels and suppressed key inflammatory mediators. Clinically, topical application of Low-Peony PDRN for 2 weeks markedly reduced transepidermal water loss in a sodium lauryl sulfate-induced skin damage model, enhancing barrier recovery (*n* = 10). Periorbital skin elasticity improved after 4 weeks of treatment (Approval No. Intertek IRB-202505-HR(1)-0001, 20 June 2025). These results indicate that Low-Peony PDRN is a promising plant-derived biomaterial of pharmacological and cosmetic significance, with potential to address skin aging.

## 1. Introduction

Skin aging is a complex biological process driven by progressive molecular, cellular, and tissue-level alterations in the epidermis and dermis [1,2,3,4]. Both intrinsic and extrinsic factors, including ultraviolet (UV) radiation, psychological stress, and environmental pollutants [5,6,7], induce excessive production of reactive oxygen species (ROS) and pro-inflammatory cytokines such as interleukin-1β (*IL-1β*), *IL-6*, *IL-8*, tumor necrosis factor-α (*TNF-α*), and cyclooxygenase-2 (*COX-2*) [8,9,10,11]. Specifically, UV-induced aging plays a major role in premature skin aging and causes distinct types of damage depending on the wavelength. UVA penetrates deeply into the dermis, leading to oxidative stress and photoaging, whereas UVB primarily affects the epidermis, causing direct DNA damage and inflammatory responses [12,13]. These UV-induced responses impair keratinocyte and fibroblast proliferation, migration, and differentiation, thereby disrupting epidermal renewal and extracellular matrix (ECM) homeostasis [14,15]. Consequently, collagen and elastin synthesis declines, leading to ECM disorganization, reduced dermal integrity, and loss of elasticity. Concurrently, the epidermal barrier deteriorates due to diminished expression of key structural proteins such as filaggrin (*FLG*), involucrin (*IVL*), and occludin (*OCLN*). These events result in increased transepidermal water loss (TEWL), dryness, and sensitivity [16,17,18]. Collectively, these molecular and structural perturbations compromise overall skin homeostasis and accelerate the visible and functional manifestations of aging.

Polydeoxyribonucleotide (PDRN), consisting of genetic components (DNA fragments) ranging from 50 to 2000 base pairs, exerts various biological effects through activation of the adenosine A2A receptor and engagement in nucleotide salvage pathways [19,20]. These effects include stimulation of cell proliferation, anti-inflammatory activity, wound healing, tissue regeneration, and angiogenesis [21,22,23,24,25]. Because of these regenerative properties, PDRN has been used to treat chronic wounds, burns, and orthopedic injuries [26]. In dermatology, PDRN has attracted attention as a potent agent that promotes dermal fibroblast activity and collagen synthesis, in addition to ECM remodeling [27,28]. These properties position PDRN as a promising candidate for applications in the pharmaceutical and cosmetic industries. Commercially available PDRN is typically derived from salmon testes. However, limitations of animal-based biomaterials and the growing demand for vegan and eco-friendly cosmetics have driven interest in plant-based alternatives [29,30]. Given their safety and eco-friendliness, plant-based biomaterials have gained considerable attention in pharmaceutical and cosmetic fields.

*Paeonia lactiflora*, commonly referred to as Chinese peony, is a traditional medicinal herb extensively used in East Asian medicine for its broad pharmacological properties, including inflammation suppression; antiviral and antimicrobial activities; regulation of immune responses; pain relief; and protective effects on the liver, heart, and kidneys [31,32]. Numerous studies over the past decades have highlighted its dermatological potential by demonstrating activities such as melanin synthesis inhibition, antioxidant effects, modulation of inflammatory pathways, and promotion of skin barrier function [33,34,35]. Consequently, *P. lactiflora* extracts have been increasingly incorporated into cosmetic formulations targeting hyperpigmentation, skin aging, and sensitive skin. Given its multifunctional bioactivities and plant-based origin, *P. lactiflora* represents a compelling candidate for the development of sustainable and effective cosmeceutical formulations. Although the biological activities of *P. lactiflora* extracts and their principal bioactive constituents, including paeoniflorin and albiflorin, have been extensively reported, biological functions at the genetic level and their relevance to skin aging remain insufficiently explored. Therefore, further biological investigation of *P. lactiflora*-derived genetic components holds considerable academic and clinical significance.

In this study, PDRN extracted from *P. lactiflora* (Peony PDRN) was investigated to confirm and optimize its biological efficacy. Accordingly, DNA fractions of varying molecular sizes were prepared and evaluated through in vitro and clinical studies to assess their anti-aging potential. Collectively, these findings aim to establish Peony PDRN as an effective plant-based biomaterial applicable in pharmaceutical and cosmetic formulations.

## 2. Results

### 2.1. Peony PDRN Is Isolated from P. lactiflora and Fractionated into Different Molecular Sizes

**Figure 1:**
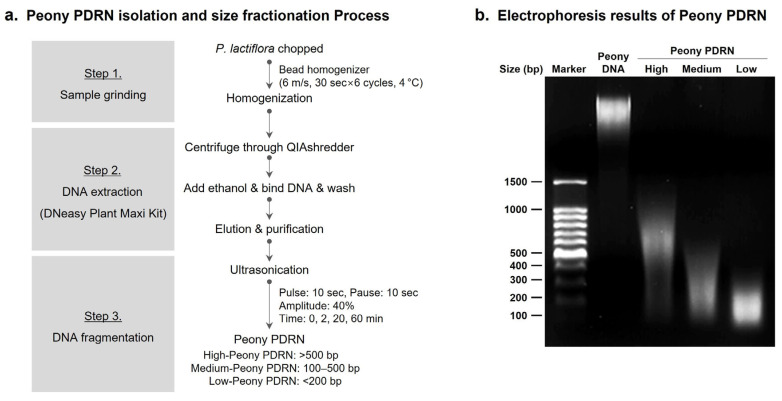
Ultrasonic homogenizer-assisted isolation and size-based purification of polydeoxyribonucleotide (PDRN) derived from *Paeonia lactiflora* (Peony PDRN). (**a**) Peony PDRN was isolated from *P. lactiflora* using bead homogenization, DNA extraction, and ultrasonic fragmentation to obtain DNA fragments of High-, Medium-, or Low-Peony PDRN. (**b**) The DNA fragment size distribution of Peony PDRN was analyzed by agarose gel electrophoresis, confirming the successful generation of high-, medium-, and low-molecular-weight fractions. DNA fragments were categorized based on size: High-Peony PDRN (>500 bp), Medium-Peony PDRN (100–500 bp), and Low-Peony PDRN (<200 bp).

Peony PDRN was extracted and fractionated to obtain DNA fragments of different molecular sizes for subsequent analyses. These fractions were successfully isolated from *P. lactiflora* through bead homogenization, DNA extraction, and ultrasonic fragmentation. Considering the potential drawbacks of chemical extraction methods—such as DNA denaturation and the presence of residual chemicals—we employed a non-chemical ultrasonication process to isolate Peony PDRN. To obtain DNA fragments of different molecular sizes, the extracted DNA was subjected to ultrasonication for 0, 2, 20, and 60 min using an ultrasonic homogenizer, enabling controlled shearing of genomic DNA (Figure 1a). DNA fragment size distribution was analyzed using agarose gel electrophoresis. As the time of ultrasonication increased, the genomic DNA exhibited progressive fragmentation. The non-sonicated sample (0 min) comprised high-molecular-weight genomic DNA, whereas samples subjected to sonication for 2 (High), 20 (Medium), and 60 min (Low) showed a gradual shift toward lower-molecular-weight fragments. After 60 min of sonication, most DNA fragments were found to be below approximately 200 bp. The fragments were categorized into three groups based on their size: High-Peony PDRN (>500 bp), Medium-Peony PDRN (100–500 bp), and Low-Peony PDRN (<200 bp) (Figure 1b). The isolated and size-fractionated Peony PDRN samples were then prepared for subsequent in vitro and in vivo analyses to evaluate their potential size-dependent biological activity.

### 2.2. Peony PDRN Enhances Proliferation of the Human Skin Cells

**Figure 2:**
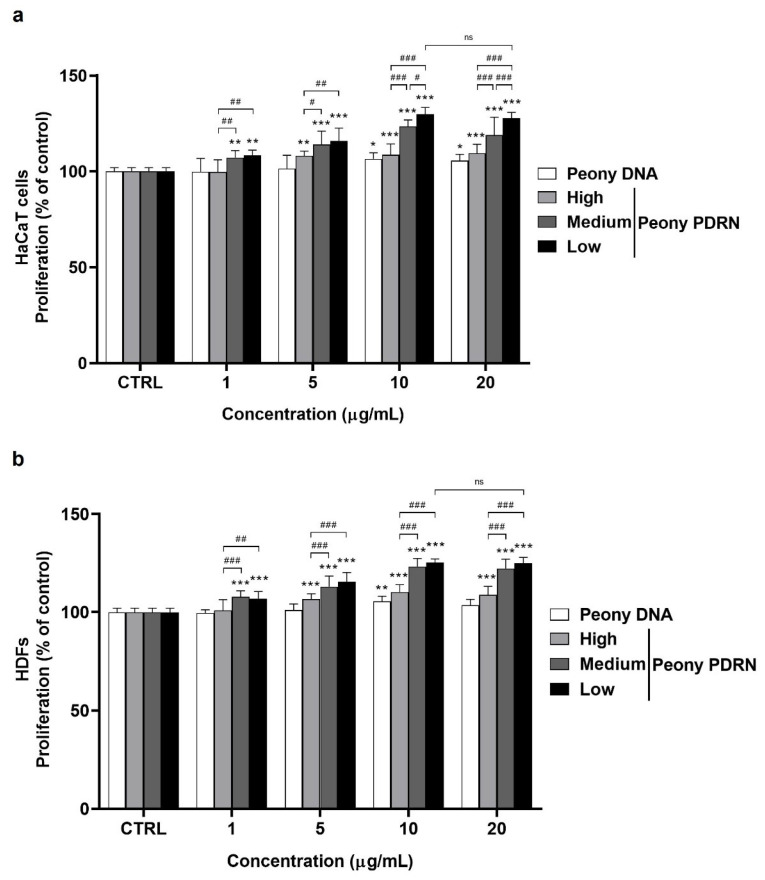
Proliferative effects of size-fractionated Peony PDRN on human keratinocytes (HaCaT cells) and human dermal fibroblasts (HDFs). HaCaT cells (**a**) and HDFs (**b**) were treated with varying concentrations (0, 1, 5, 10, and 20 μg/mL) of High-, Medium-, or Low-Peony PDRN for 48 h. Proliferation rates were determined using the water-soluble tetrazolium salt (WST) assay. Statistical significance was determined by one-way analysis of variance (ANOVA) followed by Dunnett’s post hoc test (* *p* < 0.05, ** *p* < 0.01, *** *p* < 0.001 vs. untreated control; ^#^ *p* < 0.05, ^##^ *p* < 0.01, ^###^ *p* < 0.001 vs. Peony PDRN group; ns, not significant *p* > 0.05).

To investigate the proliferative effects of size-fractionated Peony PDRN on human keratinocytes and dermal fibroblasts, a water-soluble tetrazolium salt (WST) assay was performed. Cells were treated with various concentrations (0–20 μg/mL) of High-, Medium-, or Low-Peony PDRN for 48 h. The results demonstrated a dose-dependent increase in proliferation in both human keratinocytes (HaCaT cells) and human dermal fibroblasts (HDFs). These results indicate that size-fractionated Peony PDRN exhibits no cellular cytotoxicity at designated concentrations. Importantly, a distinct size-dependent effect was also observed, with smaller DNA fragments exhibiting greater proliferative activity. Among the three Peony PDRN groups, Low-Peony PDRN showed the highest efficacy. Treatment with 10 µg/mL Low-Peony PDRN led to the strongest increase in cell proliferation, reaching 129.9% in HaCaT cells and 125.2% in HDFs compared with that in the untreated control (Figure 2). Although treatment with 20 µg/mL showed a comparable proliferative effect, the difference between 10 and 20 µg/mL was not significant. Therefore, the lower concentration (10 µg/mL) was selected for subsequent experiments to minimize potential cytotoxicity and ensure experimental consistency. These findings indicate that both the concentration and molecular weight of Peony PDRN are critical determinants of its biological activity, with lower-molecular-weight fragments exhibiting greater proliferative potential in skin-derived cell types.

### 2.3. Peony PDRN Enhances the Migration of Human Skin Cells

**Figure 3:**
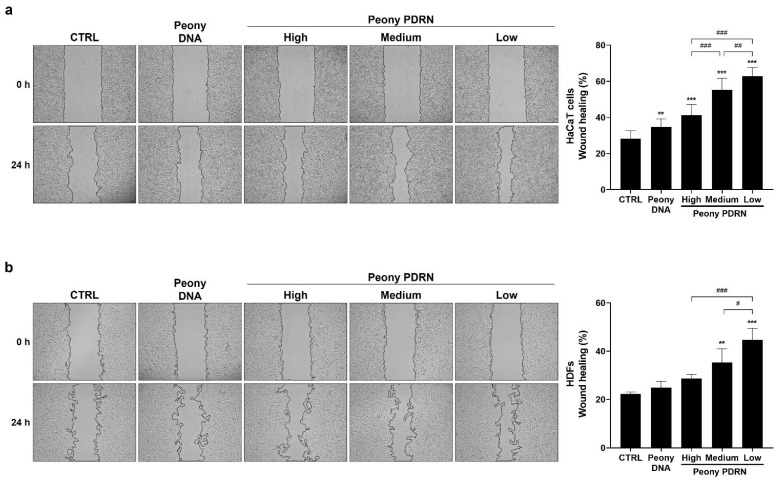
Migration effects of size-fractionated Peony PDRN on HaCaT cells and HDFs. HaCaT cells (**a**) and HDFs (**b**) were treated with 10 μg/mL of High-, Medium-, or Low-Peony PDRN for 24 h. Migration rates were determined using the wound healing assay. Statistical significance was determined by one-way ANOVA followed by Dunnett’s post hoc test (** *p* < 0.01, *** *p* < 0.001 vs. untreated control; ^#^ *p* < 0.05, ^##^ *p* < 0.01, ^###^ *p* < 0.001 vs. Peony PDRN group).

To assess the migratory effects of size-fractionated Peony PDRN on human keratinocytes and dermal fibroblasts, scratch wound healing assays were performed. Cells were treated with 10 μg/mL of High-, Medium-, or Low-Peony PDRN for 24 h. The results demonstrated a size-dependent enhancement of migratory behavior, with smaller Peony PDRN fragments, particularly Low-Peony PDRN, producing the greatest improvement in wound closure. HaCaT cell migration speed was increased 2.0-fold and that of HDF 1.8-fold compared with untreated control (Figure 3). These findings indicate that fragment size is a key determinant of Peony PDRN in wound healing efficacy, with low-molecular-weight fragments markedly enhancing wound closure compared with larger fragments, underscoring their potential utility in skin regeneration strategies.

### 2.4. Peony PDRN Enhances Expression of Genes Related to Keratinocyte Differentiation and Those Encoding ECM Proteins

**Figure 4:**
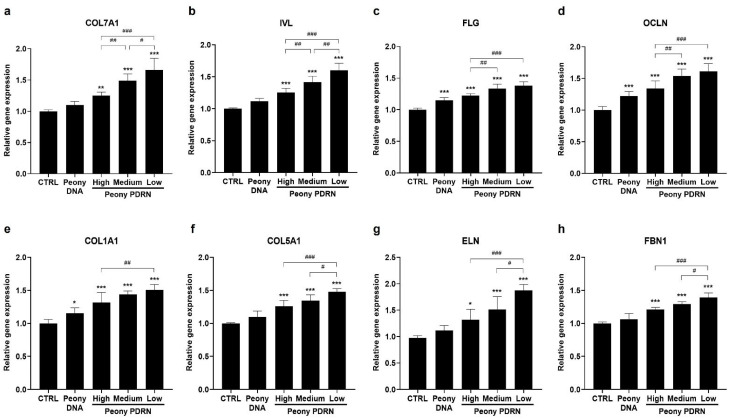
Effects of size-fractionated Peony PDRN on keratinocyte differentiation and ECM-related gene expression in HaCaT cells and HDFs. HaCaT cells were treated with 10 μg/mL of High-, Medium-, or Low-Peony PDRN for 24 h, and HDFs were treated with the same concentrations for 48 h. mRNA expression levels of *COL7A1*, *IVL*, *FLG*, and *OCLN* in HaCaT cells (**a**–**d**), and *COL1A1*, *COL5A1*, *ELN*, and *FBN1* in HDFs (**e**–**h**), were determined using real-time polymerase chain reaction (PCR). Gene expression levels are presented as fold-changes in comparison to untreated control values. Statistical significance was determined by one-way ANOVA followed by Dunnett’s post hoc test (* *p* < 0.05, ** *p* < 0.01, *** *p* < 0.001 vs. untreated control; ^#^ *p* < 0.05, ^##^ *p* < 0.01, ^###^ *p* < 0.001 vs. Peony PDRN group).

To further elucidate the biological effects of size-fractionated Peony PDRN on skin barrier function and ECM remodeling, the expression of genes related to keratinocyte differentiation and ECM synthesis was examined using real-time PCR. HaCaT cells and HDFs were treated with 10 μg/mL of High-, Medium-, or Low-Peony PDRN. The results demonstrated a size-dependent upregulation of gene expression, with smaller PDRN fragments inducing considerably higher expression levels. Specifically, Low-Peony PDRN markedly increased the expression of genes associated with keratinocyte differentiation and epidermal barrier formation, including *COL7A1*, *IVL*, *FLG*, and *OCLN*, in HaCaT cells, suggesting enhanced potential for restoring and normalizing epidermal barrier function (Figure 4a–d). In HDFs, Low-Peony PDRN also led to a markedly elevated expression of genes encoding key ECM proteins, such as *COL1A1*, *COL5A1*, *ELN*, and *FBN1*, which are critical for dermal structure and skin regeneration (Figure 4e–h). These findings indicate that as the fragment size of Peony PDRN decreases, its ability to regulate gene expression increases. Furthermore, low-molecular-weight fragments exhibited superior efficacy in promoting keratinocyte differentiation, restoring skin barrier function, and enhancing ECM remodeling relevant to skin repair.

### 2.5. Low-Peony PDRN Restores Keratinocyte Differentiation and ECM-Related Gene Expression Suppressed by UV Irradiation

**Figure 5:**
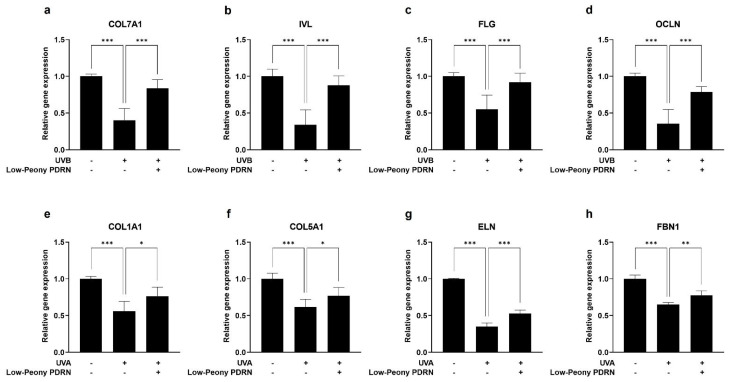
Effect of Low-Peony PDRN on keratinocyte differentiation and extracellular matrix (ECM)-related gene expression in ultraviolet (UV)-damaged HaCaT cells and HDFs. (**a**–**d**) HaCaT cells were irradiated with UVB and subsequently treated with 10 μg/mL Low-Peony PDRN for 24 h. The mRNA expression levels of *COL7A1*, *IVL*, *FLG*, and *OCLN* were measured. (**e**–**h**) HDFs were irradiated with UVA and treated with 10 μg/mL Low-Peony PDRN for 48 h. The mRNA expression levels of *COL1A1*, *COL5A1*, *ELN*, and *FBN1* were measured. Gene expression levels were determined using real-time PCR and are presented as fold-changes relative to non-irradiated controls. Statistical significance was determined by one-way ANOVA followed by Dunnett’s post hoc test (* *p* < 0.05, ** *p* < 0.01, *** *p* < 0.001 vs. UV-only group).

To investigate the restorative effects of Low-Peony PDRN on UV-induced impairment of epidermal barrier function and dermal ECM homeostasis, HaCaT cells were treated with 5 mJ/cm^2^ of UVB to induce epidermal damage, whereas HDFs were treated with 2 J/cm^2^ of UVA to mimic dermal photoaging and oxidative stress as previously described [36,37,38,39]. Following UV irradiation, cells were treated with 10 μg/mL of Low-Peony PDRN. In HaCaT cells, UVB irradiation markedly reduced the expression of genes related to keratinocyte differentiation, including *COL7A1*, *IVL*, *FLG*, and *OCLN*. However, subsequent treatment with Low-Peony PDRN effectively restored the expression levels of these genes, suggesting its potential to normalize and recover epidermal barrier function compromised by UVB exposure (Figure 5a–d). Similarly, in HDFs, UVA irradiation led to a marked decrease in the expression of genes encoding key ECM proteins, such as *COL1A1*, *COL5A1*, *ELN*, and *FBN1*, which are essential for dermal structure and elasticity. Treatment with Low-Peony PDRN successfully reversed this downregulation, indicating its ability to restore ECM-related gene expression following UVA-induced damage (Figure 5e–h). These results demonstrate that Low-Peony PDRN not only promotes gene expression under normal conditions but also restores UV-suppressed expression of keratinocyte differentiation and ECM-related genes, thereby contributing to the recovery of epidermal barrier function and dermal ECM homeostasis.

### 2.6. Low-Peony PDRN Attenuates UV-Induced Oxidative Stress by Reducing Intracellular ROS Levels in Human Skin Cells

**Figure 6:**
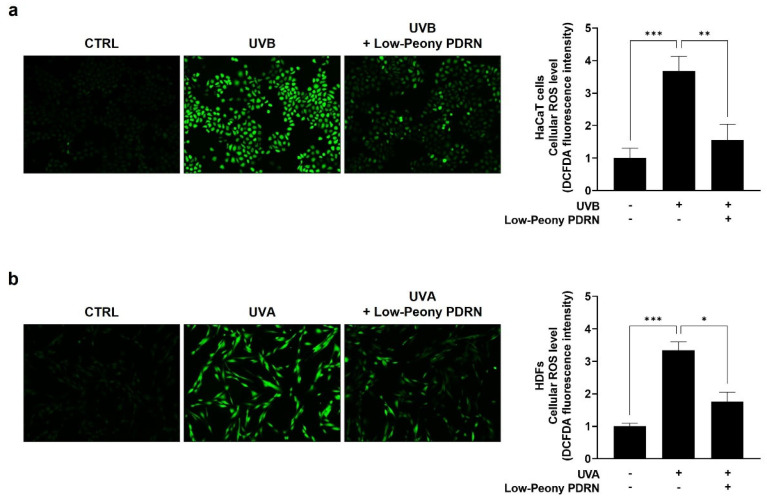
Effect of Low-Peony PDRN on UV-induced intracellular reactive oxygen species (ROS) levels in HaCaT cells and HDFs. (**a**) HaCaT cells were exposed to 5 mJ/cm^2^ of UVB and (**b**) HDFs to 2 J/cm^2^ of UVA, followed by treatment with 10 μg/mL Low-Peony PDRN for 24 h and 48 h, respectively. Intracellular ROS levels were measured using a 2′,7′-dichlorofluorescin diacetate (DCF-DA) assay. Statistical significance was determined by one-way ANOVA followed by Dunnett’s post hoc test (* *p* < 0.05, ** *p* < 0.01, *** *p* < 0.001 vs. UV-only group).

To evaluate the antioxidant potential of Low-Peony PDRN, intracellular ROS levels were measured by fluorescence intensity using a 2′,7′-dichlorofluorescin diacetate (DCF-DA) assay in UV-induced damaged skin cells. HaCaT cells were exposed to UVB and HDFs to UVA to induce epidermal and dermal oxidative stress, followed by treatment with 10 μg/mL of Low-Peony PDRN. UV irradiation considerably increased intracellular ROS levels, as reflected by increased fluorescence intensity, in both HaCaT cells and HDFs, whereas Low-Peony PDRN treatment effectively reduced ROS accumulation relative to UV-only controls (Figure 6). These results suggest that Low-Peony PDRN possesses antioxidant properties that attenuate UV-induced oxidative stress in both epidermal and dermal skin cells.

### 2.7. Low-Peony PDRN Attenuates UV-Induced Upregulation of Pro-Inflammatory Cytokines in Human Skin Cells

**Figure 7:**
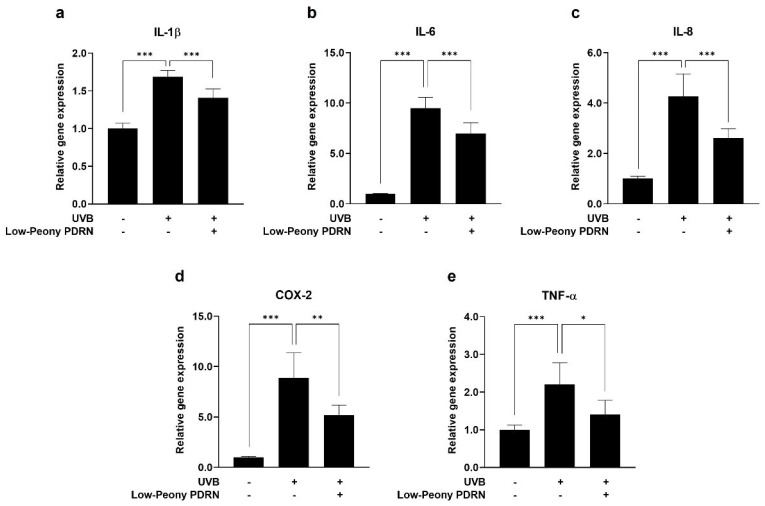
Effect of Low-Peony PDRN on UVB-induced pro-inflammatory cytokine expression in HaCaT cells. (**a**–**e**) HaCaT cells were exposed to 5 mJ/cm^2^ of UVB, followed by treatment with 10 μg/mL Low-Peony PDRN for 24 h. The mRNA expression levels of interleukin-1β (*IL-1β*), *IL-6*, *IL-8*, cyclooxygenase-2 (*COX-2*), and tumor necrosis factor-α (*TNF-α*) were measured using real-time PCR. Gene expression levels are presented as fold-changes relative to non-irradiated controls. Statistical significance was determined by one-way ANOVA followed by Dunnett’s post hoc test (* *p* < 0.05, ** *p* < 0.01, *** *p* < 0.001 vs. UV-only group).

**Figure 8:**
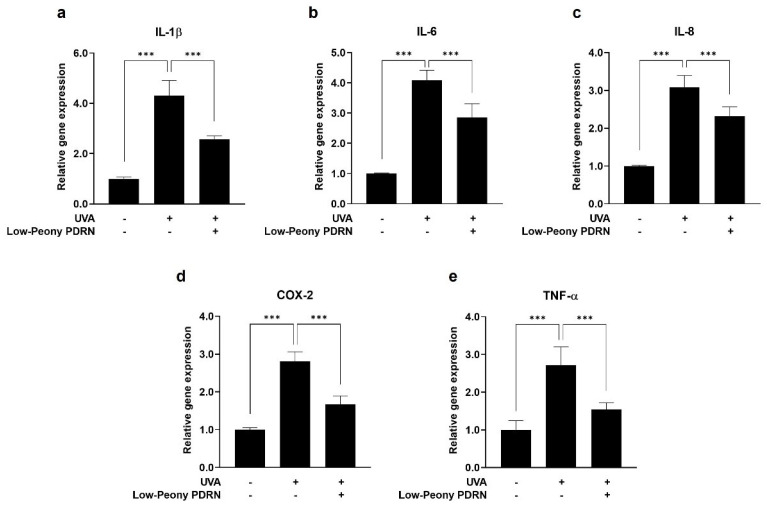
Effect of Low-Peony PDRN on UVA-induced pro-inflammatory cytokine expression in HDFs. (**a**–**e**) HDFs were exposed to 2 J/cm^2^ of UVA, followed by treatment with 10 μg/mL Low-Peony PDRN for 24 h. The mRNA expression levels of *IL-1β*, *IL-6*, *IL-8*, *COX-2*, and *TNF-α* were measured using real-time PCR. Gene expression levels are presented as fold-changes relative to non-irradiated controls. Statistical significance was determined by one-way ANOVA followed by Dunnett’s post hoc test (*** *p* < 0.001 vs. UV-only group).

To evaluate the anti-inflammatory effects of Low-Peony PDRN, the mRNA expression levels of pro-inflammatory cytokines, including *IL-1β*, *IL-6*, *IL-8*, *COX-2*, and *TNF-α*, were quantified in UV-irradiated HaCaT cells and HDFs. The cells were exposed to UVB (5 mJ/cm^2^) and UVA (2 J/cm^2^) to induce inflammatory responses, followed by treatment with 10 μg/mL of Low-Peony PDRN. UV irradiation markedly upregulated the expression of the pro-inflammatory cytokines in both cell types, whereas Low-Peony PDRN treatment considerably attenuated their expression relative to UV-only controls (Figure 7 and Figure 8). These results indicate that Low-Peony PDRN exerts anti-inflammatory effects by suppressing UV-induced inflammatory responses in both epidermal and dermal skin cells.

### 2.8. Low-Peony PDRN Improves Periorbital Skin Elasticity

Periorbital skin elasticity was evaluated in the experimental group to assess the effects of the Low-Peony PDRN formulation (Table 1). The treatment involved the application of an experimental cream containing 1% Low-Peony PDRN and a control cream without Low-Peony PDRN. Clinical concentration of Low-Peony PDRN was selected according to a previous study [40]. Periorbital skin elasticity in the experimental group showed a slight increase from a baseline R2 value of 69.22 to 69.71 after 2 weeks, which was not significant. After 4 weeks of application of the Low-Peony PDRN-containing test formulation, the mean R2 value further increased to 72.16, representing a 4.25% improvement relative to baseline. Conversely, the control group, treated with a cream without Low-Peony PDRN, did not exhibit notable changes in periorbital skin elasticity. These results indicate a slight trend toward improvement at 2 weeks, with a significant (*p* < 0.001) enhancement observed after 4 weeks of Low-Peony PDRN formulation application.

### 2.9. Low-Peony PDRN Improves TEWL Recovery After Sodium Lauryl Sulfate (SLS)-Induced Barrier Disruption

TEWL changes were evaluated in the control (without Low-Peony PDRN) and experimental groups (with 1% Low-Peony PDRN) following SLS-induced barrier disruption (Table 2 and Table 3). In the experimental group, TEWL level was increased considerably from 12.80 before SLS treatment to 17.27 after SLS treatment. Following application of the Low-Peony PDRN-containing test formulation, TEWL level was decreased to 14.93 after 1 week and further to 13.60 after 2 weeks, representing a 21.01% reduction relative to values after SLS treatment. In the control group, which received the formulation without Low-Peony PDRN, TEWL was also increased markedly from 12.96 before SLS treatment to 16.58 after SLS treatment, followed by a decrease to 15.15 after 1 week and 14.21 after 2 weeks, representing a 13.98% reduction relative to values after SLS treatment. These results indicate that topical application of the Low-Peony PDRN formulation accelerates recovery of skin barrier function more effectively compared to the control.

## 3. Discussion

This study marks a significant milestone as the first to explore *P*. *lactiflora*-derived PDRN and its potential applications. Traditionally, *P. lactiflora* is a medicinal herb renowned for its diverse pharmacological activities, including anti-inflammatory, antioxidant, and hepatoprotective effects. While its bioactive constituents have been clarified, the biological functions of its DNA-derived components, such as PDRN, remain poorly understood. Here, we present the first evidence demonstrating the novel biological efficacy of Peony-derived PDRN, highlighting its potential as a bioactive agent for skin regeneration and protection.

PDRN is a DNA-derived biopolymer known to promote tissue repair, angiogenesis, and anti-inflammatory responses. Microbial-derived PDRN, which contains smaller DNA fragments than salmon-derived PDRN, exhibits enhanced regenerative potential in skin repair and wound healing [41]. Another study identified the most effective molecular size range of PDRNs for promoting wound regeneration in a mouse skin wound model [42]. Based on these findings, we prepared Peony PDRN fractions of different molecular sizes to identify the optimal size associated with the strongest biological efficacy (Figure 1). In vitro analyses indicated that Low-Peony PDRN most effectively promoted proliferation and migration of human skin cells, as well as keratinocyte differentiation and ECM-related gene expression (Figure 2, Figure 3 and Figure 4).

Based on the observed in vitro size-dependent effects, the protective effects of Low-Peony PDRN against UV-induced skin damage were further evaluated. UV exposure, a primary driver of premature skin aging, disrupts epidermal barrier integrity, induces oxidative stress, and triggers inflammatory responses. Therefore, to evaluate the effects of Peony PDRN on early skin aging, UVB-damaged HaCaT cells (5 mJ/cm^2^) and UVA-damaged HDFs (2 J/cm^2^) were treated with Low-Peony PDRN. UV doses were chosen to induce inflammation without causing cytotoxicity [36,37,38,39]. Ultimately, Low-Peony PDRN effectively restored keratinocyte differentiation and ECM-related gene expression suppressed by UV exposure while concurrently reducing oxidative stress and suppressing pro-inflammatory cytokine expression (Figure 5, Figure 6, Figure 7 and Figure 8). Mechanistically, several intracellular signaling cascades may contribute to the in vitro effect of Low-Peony PDRN. In particular, Low-Peony PDRN may positively modulate A2A receptor activity and cytokine expression, similar to salmon-derived PDRN. Based on these insights, future studies should focus on elucidating the signaling mechanism involved in Low-Peony PDRN.

Low-molecular-weight compounds generally exhibit enhanced skin penetration. For instance, low-molecular-weight hyaluronic acid (20–300 kDa) penetrates the skin more effectively than high-molecular-weight hyaluronic acid (1000–1400 kDa) [43]. Additionally, low-molecular-weight hyaluronic acid below 50 kDa not only shows higher skin permeability but also positively influences the expression of genes involved in keratinocyte differentiation and intercellular junction formation [44]. Based on these observations, the low-molecular-weight fraction of Peony PDRN is expected to demonstrate superior skin permeation. Future studies should be conducted to evaluate the skin penetration of Peony PDRN across different molecular sizes to verify this potential.

Recent studies have increasingly explored formulations designed to improve skin penetration and stability [45,46,47,48]. Although storage at various temperatures had no significant impact on other PDRN contents for up to 12 weeks [49], evaluating stability of Low-Peony PDRN and developing suitable formulations are essential for pharmaceuticals and cosmetics. Accordingly, we plan to design optimized formulations to enhance the delivery and stability of Low-Peony PDRN in the skin. Moreover, challenges remain in improving production efficiency of Low-Peony PDRN in the field of industrial manufacturing. To address these challenges, we plan to compare our isolation method with other manufacturing processes, including low-temperature plasma [49] and microfluidizer [27] for mass production of Low-Peony PDRN.

Our clinical study demonstrated that topical application of Low-Peony PDRN effectively improved skin barrier function and dermal properties, as indicated by a marked reduction in TEWL and enhanced periorbital skin elasticity. While our clinical results showed only slight improvement in skin elasticity (*p* < 0.001), likely due to the lack of an optimized Low-Peony PDRN formulation, they are consistent with trends observed in in vitro studies on keratinocyte differentiation and ECM-related gene expression. No instances of skin irritation or adverse reactions were reported, indicating the safety of Low-Peony PDRN applied topically. However, some limitations to the clinical study warrant further trials. The primary limitation is the small sample size, comprising only 10 participants, which may restrict the statistical power and generalizability of the findings. Other limitations include the absence of active comparator and short study duration; longer trials with follow-up periods of at least >8 weeks are thus recommended. Additionally, using an optimized formulation containing Low-Peony PDRN is essential to enhance clinical efficacy. Future clinical trials should evaluate the anti-aging potential of Low-Peony PDRN for pharmacological and cosmetic applications.

## 4. Materials and Methods

### 4.1. Isolation of PDRN from P. lactiflora

The plant material was finely chopped and transferred to a 2.0 mL screw tube containing a 3 mm metal bead and then homogenized using a Bead Homogenizer (BIOPREP-24R; Allsheng, Hangzhou, China) under the following cycling conditions: 6 m/s for 30 s, with 30 s intervals, repeated for six cycles at 4 °C. The homogenized samples were centrifuged at 13,000 rpm for 15 min at 4 °C, and the supernatants were collected. DNA was extracted from the collected supernatants using the DNeasy Plant Maxi Kit (Qiagen, Hilden, Germany) according to the manufacturer’s protocol. Peony DNA in the separated tubes was sonicated using an ultrasonic homogenizer (HD 4100, Bandelin, Berlin, Germany) for 0, 2 (High), 20 (Medium), or 60 min (Low) (pulse: 10 s, pause: 10 s) at 40% amplitude to generate Peony PDRN of different molecular sizes [50]. The quality and quantity of the DNA were quantified using the Take3 Micro-Volume Plate Spectrophotometer (Epoch2, BioTek, Winooski, VT, USA). Electrophoresis was performed using 1.5% agarose gel. The molecular weights of Peony DNA and PDRN were determined using the Gel Documentation System (BIO-PRINT CX4, Vilber Lourmat, Collégien, France).

### 4.2. Cell Culture

Immortalized HaCaT cells were generously donated by Prof. SC Kim (Korea Associated Institute of Science and Technology, Daejeon, Republic of Korea), and HDFs were purchased from PromoCell (Heidelberg, Germany). The cells were cultured in Dulbecco’s modified Eagle’s medium (DMEM), supplemented with 1% penicillin–streptomycin (Welgene, Gyeongsan-si, Republic of Korea) and 10% fetal bovine serum (Thermo Fisher Scientific, Waltham, MA, USA), and incubated at 37 °C in a 5% CO_2_ incubator.

### 4.3. Cell Proliferation Assay

The proliferation of HaCaT cells and HDFs was evaluated by assessing mitochondrial enzyme activity using the WST method, which was performed using the EZ-Cytox reagent (DoGenBio, Seoul, Republic of Korea) [51]. Cells were seeded at a density of 5 × 10^3^ cells/well in 96-well plates and incubated for 24 h at 37 °C in a 5% CO_2_ incubator. The cells were treated with Peony PDRN (0, 1, 5, 10, and 20 ug/mL, High-, Medium-, or Low-Peony PDRN) for 48 h at 37 °C in a 5% CO_2_ incubator. After incubation, 10% EZ-Cytox of the media volume was added to each well and incubated for 1 h at 37 °C in a 5% CO_2_ incubator. Absorbance was measured at 450 nm using a microplate spectrophotometer (Epoch2, BioTek, Winooski, VT, USA). Cell proliferation (%) was calculated as follows: (absorbance of treated sample − blank)/(absorbance of control sample − blank) × 100.

### 4.4. Scratched Wound Healing Assay

HaCaT cells (2.5 × 10^5^ cells/well) and HDFs (1 × 10^5^ cells/well) were seeded in 24-well plates. The cells were incubated in the culture medium at 37 °C in a 5% CO_2_ incubator until they reached 90% confluency. The culture medium was removed, and after 4 h of serum starvation, a scratch was made in the center of the cells using a 200 µL pipette tip [52]. The cells were washed with Dulbecco’s phosphate-buffered saline (DPBS) to remove cell debris and then treated with serum-free medium containing 10 µg/mL of High-, Medium-, or Low-Peony PDRN for 24 h. The wound healing areas were captured using a microscope (DMi1, Leica Microsystems, Wetzlar, Germany) at 0 and 24 h and then analyzed using the ImageJ software (version 14.1, NIH, Bethesda, MD, USA). Wound healing (%) was calculated as follows: (wound area at 0 h [area pixels] − wound area at 24 h [area pixels])/wound area at 0 h, area pixels × 100 [53].

### 4.5. Real-Time Reverse Transcription PCR (qRT-PCR)

RNA was extracted from HaCaT cells and HDFs using the RNeasy Mini Kit (Qiagen, Hilden, Germany) according to the manufacturer’s protocol. The total RNA (1 μg) was reverse-transcribed into cDNA using the PrimeScript™ RT Reagent Kit with gDNA Eraser (TaKaRa Bio, Kusatsu, Japan) and the Applied Biosystems MiniAmp™ Plus Thermal Cycler (Thermo Fisher Scientific, Waltham, MA, USA). Real-time PCR was performed by adding 10 μL of Applied Biosystems PowerUp™ SYBR™ Green Master Mix (Thermo Fisher Scientific, Waltham, MA, USA), 10 pmol/μL of forward primer, 10 pmol/μL of reverse primer, and 1 μL of cDNA to RNase-free distilled water, for a final volume of 20 μL. Gene amplification was performed using the Applied Biosystems QuantStudio™ 3 Real-Time PCR system (Thermo Fisher Scientific, Waltham, MA, USA) under the following thermal cycling conditions: 40 cycles of 2 min at 50 °C, 2 min at 95 °C, 15 s at 95 °C, 15 s at 60 °C, and 1 min at 72 °C. Relative gene expression was analyzed using the 2^−ΔΔCt^ method [54]. The primers used for gene amplification and their sequences are presented in Table 4.

### 4.6. UVA/UVB Irradiation

To prepare HaCaT cells and HDFs for UV irradiation, the cells were cultured in DMEM supplemented with 1% penicillin–streptomycin and 10% fetal bovine serum. The culture medium was removed, and the cells were washed with DPBS to prepare HaCaT cells and HDFs for UV irradiation. DPBS was added to the cells, and the plate lids were removed for UV exposure. UV irradiation was performed using a Bio-Sun UV irradiation system (Vilber Lourmat, Collégien, France). HaCaT cells were irradiated with 5 mJ/cm^2^ UVB, whereas HDFs were irradiated with 2 J/cm^2^ UVA for the experiment [36,37,38,39].

### 4.7. Measurement of Intracellular ROS Levels

Intracellular ROS levels were measured using a cellular ROS assay kit (Abcam, Cambridge, UK) based on the principle of staining with the oxidation-sensitive fluorescent dye DCF-DA [55]. The cells were treated with UV and 10 µg/mL Peony PDRN and incubated for 24 h at 37 °C in a 5% CO_2_ incubator. Following incubation, the cells were stained with 10 μM DCF-DA at 37 °C for 45 min in the dark in a 5% CO_2_ incubator and then washed three times with a washing buffer. Fluorescent images were captured and analyzed using the live fluorescent cell movie analyzer (JuLI™ FL, NanoEntek, Seoul, Republic of Korea). Quantification was performed using ImageJ software (version 14.1, NIH, Bethesda, MD, USA).

### 4.8. Clinical Study Design

The clinical trial (Approval No. Intertek IRB-202505-HR(1)-0001, 20 June 2025) was conducted for 4 weeks with 10 healthy Korean participants aged 20–59 years (mean age, 41.6 ± 8.5 years). Participants had no underlying skin diseases. Individuals with a history of systemic steroid or retinoid treatments within 6 months, topical steroid use for more than 1 month, or cosmetic procedures (including botulinum toxin, fillers, and dermabrasion) within 6 months were excluded.

### 4.9. Clinical Evaluation Procedures

All measurements were conducted under strictly controlled environmental conditions to ensure the consistency and reliability of the results. Participants washed their faces with a standardized cleanser provided by the research team and were allowed to acclimate quietly in a temperature- and humidity-controlled room (22 ± 2 °C, 50 ± 10% relative humidity) for 30 min before measurements were taken. This standardized approach ensured that all individuals were evaluated under identical conditions.

### 4.10. Measurement of Skin Elasticity

Periorbital skin elasticity was measured using a Cutometer dual MPA580 (Courage + Khazaka, Köln, Germany). The overall elasticity parameter (R2, %) was used as the primary outcome measure. Each site was measured three times, and the mean value was calculated for analysis. The Low-Peony PDRN-containing test formulation was applied to the test area twice daily (morning and evening) after facial cleansing for 4 weeks. Measurements were performed at baseline, after 2 weeks of formulation use, and after 4 weeks of formulation use to evaluate the effects on skin elasticity.

### 4.11. Measurement of TEWL

To assess skin barrier function, skin irritation was induced on the upper arm by applying 25 μL of 1% SLS solution using an IQ patch chamber (Chemotechnique Diagnostics, Vellinge, Sweden) for 24 h [56]. TEWL was measured with a Tewameter TM300 (Courage + Khazaka, Köln, Germany) and expressed in g/h/m^2^. The test and control areas on the upper arm were treated with the experimental formulation (containing Low-Peony PDRN) and the control formulation (without Low-Peony PDRN), respectively, twice daily (morning and evening) for 2 weeks. Measurements were taken before SLS treatment, immediately after SLS treatment, and after 1 and 2 weeks of formulation application to evaluate the recovery of skin barrier function.

### 4.12. Statistical Analyses

For the in vitro tests, the data are presented as the mean ± standard deviation (SD), with a minimum of three independent experiments conducted. Statistical analysis was performed using the GraphPad Prism 8 (GraphPad Software, San Diego, CA, USA). Significance was determined using one-way analysis of variance (ANOVA) followed by Dunnett’s post hoc test. A *p*-value of <0.05 was considered statistically significant.

For clinical efficacy assessments, the data are presented as the mean ± SD. Changes in skin elasticity and TEWL over time were analyzed using repeated-measures ANOVA with Bonferroni post hoc correction, and between-group differences in TEWL were assessed using independent *t*-tests. Statistical analysis was performed using SPSS Statistics 26 Standard (IBM, New York, NY, USA). A *p*-value of <0.05 was considered statistically significant.

## 5. Conclusions

Our findings support the application of Peony PDRN, particularly its low-molecular-weight fraction (Low-Peony PDRN), as a plant-derived biomaterial with notable regenerative, barrier-enhancing, and anti-aging properties. The upregulation of genes associated with keratinocyte differentiation and ECM remodeling under UV-induced photodamage highlights the potential of Low-Peony PDRN to restore epidermal and dermal homeostasis. Reductions in UV-induced ROS levels and suppression of pro-inflammatory cytokine expression further support its protective effects in aging and environmentally stressed skin. Additionally, clinically observed improvements in TEWL and periorbital skin elasticity further substantiate its functional efficacy as an active ingredient in anti-aging skincare. Overall, these results highlight the potential of Low-Peony PDRN as a novel biomaterial for attenuating skin aging, providing a foundation for its further development and application in pharmacological and cosmetic formulations.

## Figures and Tables

**Table 1:** Changes in periorbital skin elasticity (R2) at baseline, 2 weeks, and 4 weeks.

Variable	Group	Measurement (M ± SD)			*p*-Value	
		Baseline	2 Weeks	4 Weeks	Baseline-2 Weeks	Baseline-4 Weeks
R2	Control (n = 10)	69.36 ± 8.02	69.57 ± 8.17	69.59 ± 8.60	-	-
	Experimental (n = 10)	69.22 ± 7.91	69.71 ± 7.93	72.16 ± 8.24	-	0.000 ***

M, mean; SD, standard deviation; *** *p* < 0.001 vs. baseline.

**Table 2:** Changes in TEWL after one and two weeks of test formulation use.

Group	TEWL (M ± SD)				*p*-Value	
	Before SLS	After SLS	1 Week	2 Weeks	1 Week After SLS	2 Weeks After SLS
Control (n = 10)	12.96 ± 1.45	16.58 ± 1.58	15.15 ± 1.17	14.21 ± 1.09	0.008 **	0.002 **
Experimental (n = 10)	12.80 ± 1.67	17.27 ± 1.93	14.93 ± 1.66	13.60 ± 1.48	0.000 ***	0.000 ***

TEWL, transepidermal water loss; M, mean; SD, standard deviation; SLS, sodium lauryl sulfate; ** *p* < 0.01, *** *p* < 0.001 vs. after SLS.

**Table 3:** Comparisons of reduction rate in TEWL at one and two weeks after SLS treatment.

Time Point (vs. After SLS)	Reduction Rate (%)		*p*-Value
	Control (n = 10)	Experimental (n = 10)	
1 week	8.40	13.43	0.035 *
2 weeks	13.98	21.01	0.026 *

TEWL, transepidermal water loss; SLS, sodium lauryl sulfate; * *p* < 0.05 vs. control. Improvement rate (%) = (after test formulation − after SLS treatment)/after SLS treatment × 100.

**Table 4:** The primer sequences for qRT-PCR.

Gene Name		Primer Sequence (5′ → 3′)
*COL1A1*	Forward	CAT AAA GGG TCA CCG TGG CT
	Reverse	GGG ACC TTG TTC ACC AGG AG
*COL5A1*	Forward	ACC ACC AAA TTC CTC GAC C
	Reverse	CCT CAA ACA CCT CCT CAT CC
*COL7A1*	Forward	GTT GGA GAG AAA GGT GAC GAG G
	Reverse	TGG TCT CCC TTT TCA CCC ACA G
*ELN*	Forward	TGT CCA TCC TCC ACC CCT CT
	Reverse	CCA GGA ACT CCA CCA GGA AT
*FBN1*	Forward	GGA TAC ACA GGT GAT GGC TTC AC
	Reverse	GTC GCA TTC ACA GCG GTA TCC T
*FLG*	Forward	AGG CTC CTT CAG GCT ACA TTC
	Reverse	CAG GAG AGT AGA CAT CTT TTG GCA
*IVL*	Forward	TAA CCA CCC GCA GTG TCC AG
	Reverse	ACA GAT GAC GGG CCA CCT A
*OCLN*	Forward	TTT GTG GGA CAA GGA ACA CA
	Reverse	ATG CCA TGG GAC TGT CAA CT
*IL-1β*	Forward	AAA CAG ATG AAG TGC TCC TTC CAG G
	Reverse	TGG AGA ACA CCA CTT GTT GCT CCA
*IL-6*	Forward	GCC TTC GGT CCA GTT GGC TT
	Reverse	GCA GAA TGA GAT GAG TTG TC
*IL-8*	Forward	ACT GTG TGT AAA CAT GAC TTC C
	Reverse	CAC TGG CAT CTT CAC TGA TTC T
*TNF-α*	Forward	CTT GTT CCT CAG CCT CTT C
	Reverse	GCT GGT TAT CTC TCA GCT C
*COX-2*	Forward	GAA TGG GGT GAT GAG CAG TT
	Reverse	CAG AAG GGC AGG ATA CAG C
*GAPDH*	Forward	ACC CAC TCC TCC ACC TTT GA
	Reverse	CTG TTG CTG TAG CCA AAT TCG T

qRT-PCR, Real-Time Reverse Transcription Polymerase Chain Reaction; *COL1A1*, Collagen Type I Alpha 1 Chain; *COL5A1*, Collagen Type V Alpha 1 Chain; *COL7A1*, Collagen Type VII Alpha 1 Chain; *ELN*, Elastin; *FBN1*, Fibrillin 1; *FLG*, Filaggrin; *IVL*, Involucrin; *OCLN*, Occludin; *IL-1β*, Interleukin-1 Beta; *IL-6*, Interleukin-6; *IL-8*, Interleukin-8; *TNF-α*, Tumor Necrosis Factor Alpha; *COX-2*, Cyclooxygenase-2; *GAPDH*, Glyceraldehyde-3-Phosphate Dehydrogenase.

## Data Availability

The original contributions presented in this study are included in the article. Further inquiries can be directed to the corresponding author.

## Abbreviations

The following abbreviations are used in this manuscript:

COL1A1	Collagen, type I, alpha 1
COL5A1	Collagen, type V, alpha 1
COL7A1	Collagen, type VII alpha 1
COX-2	Cyclooxygenase-2
DCF-DA	2′,7′-dichlorofluorescin diacetate
DEJ	Dermal-epidermal junction
DMEM	Dulbecco’s modified Eagle’s medium
ECM	Extracellular matrix
ELN	Elastin
FBN1	Fibrillin1
FLG	Filaggrin
GAPDH	Glyceraldehyde 3-phosphate dehydrogenase
HaCaT cells	Human keratinocytes
HDFs	Human dermal fibroblasts
IL-1β	Interleukin-1 beta
IL-6	Interleukin 6
IL-8	Interleukin-8
IVL	Involucrin
OCLN	Occludin
PDRN	Polydeoxyribonucleotide
ROS	Reactive oxygen species
SLS	Sodium lauryl sulfate
TEWL	Transepidermal water loss
TNF-α	Tumor necrosis factor-alpha
WST	Water-soluble tetrazolium salt
